# The Effect of Traction before Closed Reduction in Patients with Developmental Dysplasia of the Hip

**DOI:** 10.3390/children9091325

**Published:** 2022-08-31

**Authors:** Sanjiv S. G. Gangaram-Panday, Suzanne de Vos-Jakobs, Max Reijman

**Affiliations:** Department of Orthopaedics and Sports Medicine, Erasmus MC University Medical Center Rotterdam, P.O. Box 2040, 3000 CA Rotterdam, The Netherlands

**Keywords:** traction, developmental dysplasia of the hip, closed reduction, avascular necrosis

## Abstract

Developmental dysplasia of the hip (DDH) with a dislocated hip can be treated with traction before closed reduction (CR). Currently, there is insufficient evidence supporting the use of preoperative traction treatment for a successful CR. The objective of this study was to determine the effect of preoperative traction on the success rate of primary CR in DDH patients with dislocated hips. A retrospective pair-matched study was performed in DDH patients with dislocated hips. Patients with preoperative traction treatment prior to primary CR were matched (based on age and the severity of DDH on the radiograph) to patients without preoperative traction treatment. The primary outcome was the presence or absence of maintained reduction after three weeks. A match was found for 37 hips, which resulted in the inclusion of 74 hips. No significant difference was found in the number of successful reductions after three weeks between the traction group and the control group (31 vs. 33 hips, *p* = 0.496). Traction treatment did not significantly improve the short-term or mid-term outcomes for closed reduction. Based on these results, we suggest that traction treatment should not be used as standard care for dislocated hips in DDH.

## 1. Introduction

Developmental dysplasia of the hip (DDH) is the most common musculoskeletal disorder in infants and young children [1]. DDH includes a wide spectrum of developmental disorders of the hip, varying from stable dysplastic hips to unstable or dislocated hips [2].

Currently, hip dislocation due to DDH is first treated with a Pavlik harness [3]. If the Pavlik harness fails, the next step is closed reduction (CR) and the application of a spica cast under general anesthesia [4]. CR is considered successful when the femoral head is correctly positioned in the acetabulum and remains reducedduring follow-up. If dislocation persists or redislocation occurs, an open reduction can be performed. Open reduction has more complications than CR, and should preferably be avoided [5].

As with all medical interventions, CR has a risk of complications, e.g., redislocation (8–40%) and avascular necrosis (AVN) of the femoral head (10%) [6,7,8].

When the hip has a limited range of motion, or when the femoral head has migrated proximally, traction treatment prior to CR can be used to improve the success rate and to reduce the incidence of AVN [6,8,9,10,11]. During this treatment, the hips are gradually reduced via traction and abduction. The range of motion of the hip is improved as the muscles and ligaments are stretched due to traction. There is a wide variation in traction methods and duration [12].

Whether or not traction treatment improves the success rate of CR and reduces the incidence of AVN, it remains controversial. Previous studies have shown no clinically relevant difference in AVN or success rate of CR [7,12,13,14]. Currently, there is no consensus in the literature for whether traction treatment should be used as part of standard care.

The primary objective of this study was to determine the effect of traction treatment on the success rate of a primary CR, defined as maintained reduction, in DDH patients with dislocated hips. Secondary, the effect of traction on (1) long-term redislocation, (2) the number of adductor tendon tenotomies at primary CR, (3) the development of AVN, (4) residual dysplasia, and (5) the improvement of acetabular development is evaluated.

## 2. Materials and Methods

### 2.1. Study Design

A retrospective pair-matched study was performed in DDH patients with dislocated hips, treated with CR from 2010 to 2018 at the Department of Pediatric Orthopaedics at our hospital. The Medical Ethics Committee of our institution provided a waiver of approval for this study (MEC-2018-1525).

The inclusion criteria were (1) DDH with 1 or 2 dislocated hips, (2) primary CR with or without preoperative traction treatment, (3) spica cast for three months (range, 10–14 weeks) and (4) a follow-up of minimally three weeks. Patients were excluded in cases of (1) teratologic dislocation, (2) neuromuscular disease, (3) previous CR, (4) incomplete data, (5) incomplete traction treatment, or (6) a combination of CR with pelvic or femoral surgery.

Two patient groups were identified: (1) traction treatment prior to CR and (2) direct CR (control group). Whether or not traction treatment was started was the physician’s choice, based on clinical and radiographic findings, such as the Ortolani test, limited abduction, and radiographic presence of a neoacetabulum.

Traction treatment consisted of two weeks of traction in a clinical setting. Vertical traction (90° hip flexion) was used for patients under six months of age, and horizontal traction (hip extension), for patients older than six months. Three age groups were defined, based on the type of traction (horizontal or vertical) and hip development: 0–6 months, 6–9 months, and older than 9 months. The initial anteroposterior (AP) pelvic radiograph was evaluated in each patient for the severity of the dislocation. This was categorized based on the presence of a neoacetabulum. Patients were matched by age group and severity of the dislocation. Bilateral dislocated hips were separately matched for both sides. The investigator who matched the cases was blinded to the outcome.

### 2.2. Outcome Measures

The primary outcome was a successful CR, which is defined as a maintained reduction at three weeks after the CR procedure. The position of the hip at three weeks was evaluated via transinguinal ultrasound [15]. A subgroup analysis was performed on the primary outcome in the three age groups.

The secondary outcome measures were (1) adductor tendon tenotomy during primary CR, (2) redislocation at six months after CR, (3) the presence of AVN, and (4) residual dysplasia at one year (a range of 9–18 months) and two years (a range of 21–30 months) of follow-up (5), and an improvement in acetabular development at one year and two years of follow-up. No subgroup analysis was performed on the secondary outcome measures. 

AVN was defined using the Salter criteria and classified as a dichotomous outcome [16]. Residual dysplasia was defined as an acetabular index (AI) of 25 degrees or higher. Improvement of the acetabular development (the progression of AI) was calculated as the difference between the AI at baseline and the AI at the given times at follow-up. This measure can indicate the speed of improvement of the acetabulum.

### 2.3. Data Extraction

Baseline characteristics and outcome data were extracted from the medical charts, radiographic images, and surgery reports, using the hospital information system (PDMS, Picis Clinical Solutions, Wakefield, USA; Hix, ChipSoft B.V., Amsterdam, The Netherlands). When in doubt, a second opinion from a pediatric orthopedic surgeon was requested.

### 2.4. Statistical Analysis

Categorical variables are presented as numbers and percentages. Continuous variables were tested using the Shapiro–Wilk test for normality. If data showed a normal distribution, these were presented as mean and standard deviation (SD). Otherwise, data were presented as median and interquartile range (IQR). After the data were matched, the number of successful CR procedures and the number of adductor tendon tenotomies were analyzed using McNemar’s test. Other secondary outcomes were tested using the chi-squared test or Fisher’s exact test if the data were binary. Continuous variables were analyzed with either an unpaired *t*-test or the Mann–Whitney *U* test. Significance was set at a *p* value of <0.05. IBM SPSS Statistics 24 (IBM Corp., Armonk, NY, USA) was used for statistical analysis.

## 3. Results

### 3.1. Patient Inclusion

**Figure 1:**
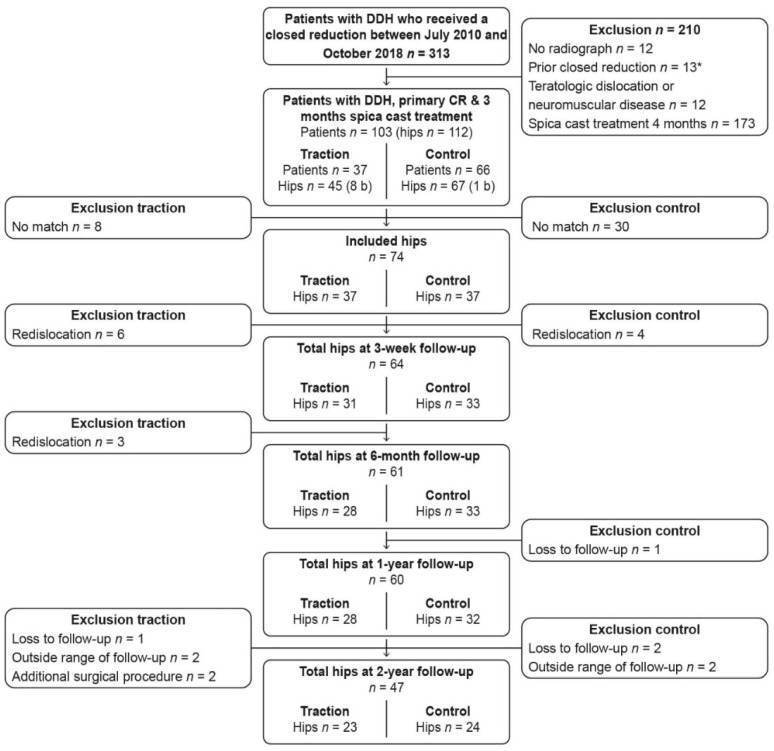
Flowchart of patient inclusion and follow-up. DDH = developmental dysplasia of the hip; CR = closed reduction; *n* = number; b = bilateral. * Patients with a closed reduction in the medical history were excluded.

From July 2010 to October 2018, a total of 335 patients were treated with CR. One hundred and three patients met the inclusion and exclusion criteria, of which 37 had preoperative traction (Figure 1). For these 37 hips, 37 matching control hips were found, which resulted in a total of 74 hips.

### 3.2. Baseline Characteristics

Of the included patients, 89% were female and had a median (IQR) age of 31.8 (22.2–37.3) weeks at CR (Table 1). The presence of a neoacetabulum was seen on 56 AP pelvic radiographs in both groups. Additional baseline characteristics can be found in Table 1.

### 3.3. Primary and Secondary Outcomes

A total of 64 (86.5%) hips were successfully reduced after three weeks, with 10 (13.5%) redislocations occurring (Table 2). There was no significant difference in the success rates between the traction group and the control group (84% and 89%, *p* = 0.496) at three weeks follow-up.

In the age group 0–6 months, three (8%) redislocations were observed in the traction group, and one (3%) in the control group. For the age group 6–9 months, these numbers were two (5%) in the traction group and two (5%) in the control group. In the age group 9–21 months (the oldest match was 21 months), in both groups one (3%), redislocation was observed. Because of the small sample sizes, no statistical significance could be calculated for the subgroups.

The number of redislocations within 6 months after CR did not differ significantly between the two groups (*p* = 0.127) (Table 2).

The other secondary outcomes (number of adductor tendon tenotomies, AVN, residual dysplasia, and acetabular improvement) showed no significant differences between the traction group and the control group (Table 3).

At one year, 60 hips, and at two years, 49 hips, were in the follow-up. The total percentage of AVN was 22% at one year and 14% at two years of follow-up, no significant difference between the traction and control groups was found (Table 3).

## 4. Discussion

### 4.1. Redislocations

In this study, the additional value of preoperative traction treatment for stable closed reduction in DDH was evaluated. The main objective was to determine whether traction treatment improves the success rate of CR. No significant difference in maintained reduction was found between the traction and the control group at three weeks and at six months of follow-up.

In this study, we chose dislocation at three weeks after CR as the primary outcome, because most redislocations occur within the first three weeks after CR, based on our clinical experience. The literature suggests that the majority of redislocations can be expected within two weeks after CR [17]. We hypothesized that the effect of traction treatment does not last longer than three weeks. Stretched ligaments and tendons will adapt to their preferred lengths rapidly, but this is most likely within three weeks after the discontinuation of traction.

At six months follow-up, more dislocations were reported in the traction group when compared to the control group (not significant, *p* = 0.127). We have no clear explanation for this finding, but this might be due to baseline differences that could not be identified in this retrospective study.

### 4.2. Adductor Tendon Tenotomies, AVN, and Residual Dysplasia

We expected to find a decrease in adductor tendon tenotomies in the traction group, due to the gradual stretching of the tendons and muscles, including adductors. However, no significant difference was found between the two groups. This could be caused by hip rigidity in the traction group, requiring both traction and tenotomy. The effect of traction treatment could be limited, necessitating additional tenotomy.

One of the main reasons for commencing preoperative traction treatment is to reduce the risk of AVN. In our study, no difference between the groups was seen in AVN incidence at the one-year and two-year follow-ups. Previous research has shown that AVN at these young ages may not deteriorate any further, and can stay clinically insignificant [18]. To determine more clearly what type of AVN our patients will develop, and what the clinical importance will be, an evaluation at a later age will be needed [19]. The outcome of this study is similar to the results of two meta-analyses, in which no significant difference in AVN rate was found when preliminary traction treatment was applied [12,14].

No differences were reported in residual dysplasia and the improvement in AI between the traction and control groups; therefore, we can conclude that the AI improves at a similar pace.

### 4.3. Limitations

The main limitation of our study is selection bias, as the decision to give traction treatment was made by the primary physician. This decision was based on clinical findings (e.g., the range of motion and Ortolani) and the radiograph. There was a significant difference in range of motion and the Ortolani test at baseline between the two groups. This implies that hip rigidity was linked to traction treatment, causing these differences between the groups. These parameters could not be included in the matching procedure of the hips, due to a relatively high rate of missing data.

Secondly, we chose to classify and match the hips in the study population based on the presence of a neoacetabulum on pelvic radiographs. Although more measurements based on the radiograph and clinical assessment could be included in the matching method, these measurements provided insufficient information on the initial state of the hip. We believe that the presence or absence of a neoacetabulum provides most information on the duration of dislocation, stiffness, and chances of successful CR.

The effect of traction treatment on the number of open reductions was not investigated in this study. A recent study concluded that traction treatment does not reduce the cases of open reduction [20]. Future prospective randomized controlled trials (RCT) should include both closed and open reduction.

### 4.4. Interpretation of Findings

Currently, in most studies, successful CR is defined as a femoral head that is reduced in the acetabulum during the procedure and maintained in this position during follow-up [7,12,21]. We strongly believe that the effect of preoperative traction treatment is only present in the first days to weeks after the treatment; this is confirmed by our results. Therefore, the effect of traction treatment on the reduction can be determined in an early stage following CR. Inadequate acetabular remodeling can lead to instability and redislocations, but this occurs later during follow-up and is not affected by traction treatment.

The relation between traction treatment and the risk of AVN is difficult to investigate, as AVN is a multifactorial problem that can be provoked at all steps of treatment (e.g., Pavlik, traction, CR, and spica cast). Additionally, AVN can present multiple years after an intervention, making causality hard or impossible to prove, even with an RCT. As traction treatment is a challenging process for both child and parent, and the value is questionable, we do not advise traction treatment for this purpose anymore.

## 5. Conclusions

In this study, we did not identify any short-term or long-term benefits of traction treatment.

Previous comparative studies showed no benefit for traction treatment in achieving a higher success rate of CR, which is in line with our findings [7,12]. There are no studies comparing traction treatment to a control group that are in favor of traction treatment.

Based on these results, we suggest that traction treatment should not be used as standard care for dislocated hips in DDH. These results should be confirmed in prospective RCTs.

## Figures and Tables

**Table 1:** Baseline characteristics.

		Traction Group H37/P35	Control Group H37/P37	*p* Value
Age at intervention (weeks)		32.4 (23–36.7)	31.7 (20.9–37.3)	0.474
Sex	Female	32 (86.5)	34 (91.9)	0.687
Family history of DDH	+	13 (39.4) **	12 (32.4)	0.504
Breech position	+	7 (21.9) ^^^	12 (35.3) ^^^	0.287
Side	Left	23 (65.7)	28 (75.7)	0.667
	Right	5 (14.3)	8 (21.6)	
	Bilateral	7 (20)	1 (2.7)	0.031
AIF difference (degrees)		35 (15–45) *	20 (10–31.3) ^#^	0.026
Ortolani	+	6 (17.6) ^^^	20 (64.5) ^#^	<0.001
AI baseline (degrees)		39.1 (±4.4)	37.4 (±4.4)	0.148
Pavlik		25 (67.6)	21 (56.8)	0.454

DDH = developmental dysplasia of the hip; AIF = abduction in flexion; AI = acetabular index; H = hips; P = patients; Age and AIF differences presented as median (interquartile range); AI baseline presented as mean (±standard deviation); Categorical values are presented as number (%); AIF difference is observed in unilateral dislocated hips; Two bilateral dislocated hips in the traction group were matched for both sides, the remaining bilateral patients were matched for one side; Missing data * *n* = 1; ** *n* = 2; ^^^ *n* = 3; ^#^ *n* = 6.

**Table 2:** Primary and secondary outcomes of redislocation.

		Traction (*n* = 37)	Control (*n* = 37)	*p* Value
Successful CR at 3 weeks		31 (83.8)	33 (89.2)	0.496
Redislocations at 3 weeks	Age group			
	0–6 m: *n* = 26	3 (8.1)	1 (2.7)	-
	6–9 m: *n* = 34	2 (5.4)	2 (5.4)	-
	9–21 m: *n* = 14	1 (2.7)	1 (2.7)	-
Redislocations at 6 months		9 (24)	4 (10.8)	0.127

CR = closed reduction; *n* = number; m = months; Data is presented as number (%).

**Table 3:** Secondary outcomes for adductor tendon tenotomy, AVN, and residual dysplasia.

	Total Hips	Traction	Control	*p* Value
Adductor tendon tenotomy	H74	13 (35.1)	11 (29.7)	0.824
AVN at 1 year	H60	6 (21.4)	7 (21.9)	1.000
AVN at 2 years	H49	4 (16.7)	3 (12)	0.702
Residual dysplasia (AI > 25°) at 1 year	H58	22 (78.6)	19 (63.3)	0.25
Residual dysplasia (AI > 25°) at 2 years	H49	16 (66.7)	13 (52)	0.296
Progression AI at 1 year	H58	10.9 (±5.1)	9.9 (±4.8)	0.432
Progression AI at 2 years	H49	13.5 (±7.4)	12.9 (±4.2)	0.771

AVN = avascular necrosis; AI = acetabular index; H = hips. At one year, 60 hips (28 traction, 32 control), and at two years, 49 hips (24 traction; 25 control) were in the follow-up. AI in degrees presented as mean (±SD); Categorical variables are presented as numbers (%).

## Data Availability

The data presented in this study are available on request from the corresponding author.
